# Transcriptome-wide m6A methylation in natural yellow leaf of *Catalpa fargesii*

**DOI:** 10.3389/fpls.2023.1167789

**Published:** 2023-06-19

**Authors:** Yu Zhang, Junhui Wang, Wenjun Ma, Nan Lu, Pengyue Fu, Yingying Yang, Linjiao Zhao, Jiwen Hu, Guanzheng Qu, Nan Wang

**Affiliations:** ^1^ State Key Laboratory of Tree Genetics and Breeding, Research Institute of Forestry, Chinese Academy of Forestry and Northeast Forestry University, Beijing, China; ^2^ Hekou Yao Autonomous County Forestry and Grassland Bureau, Hekou, China

**Keywords:** *Catalpa fargesii*, CfALKBH5, Epitranscriptomics, RNA methylation, N6-methyladenosine, yellow-green leaf

## Abstract

N6-methyladenosine (m6A) is the most abundant internal modification in eukaryotic messenger RNA, and involved in various biological processes in plants. However, the distribution features and functions of mRNA m6A methylation have been poorly explored in woody perennial plants. In this study, a new natural variety with yellow-green leaves, named Maiyuanjinqiu, was screened from the seedlings of *Catalpa fargesii*. Based on the preliminary experiment, the m6A methylation levels in the leaves of Maiyuanjinqiu were significantly higher than those in *C. fargesii*. Furthermore, a parallel analysis of m6A-seq and RNA-seq was carried out in different leaf color sectors. The result showed that m6A modification were mostly identified around the 3’-untranslated regions (3’-UTR), which was slightly negatively correlated with the mRNA abundance. KEGG and GO analyses showed that m6A methylation genes were associated with photosynthesis, pigments biosynthesis and metabolism, oxidation-reduction and response to stress, etc. The overall increase of m6A methylation levels in yellow-green leaves might be associated with the decreased the expression of RNA demethylase gene *CfALKBH5*. The silencing of *CfALKBH5* caused a chlorotic phenotype and increased m6A methylation level, which further confirmed our hypothesis. Our results suggested that mRNA m6A methylation could be considered as a vital epigenomic mark and contribute to the natural variations in plants.

## Introduction

Leaf color variation is a widespread phenomenon in nature, and has been widely used as an ornamental trait in plant kingdoms. In recent years, researchers have drawn attention to the study of leaf color mechanism, because they are excellent materials for investigating pigment metabolism, chloroplast development, photosynthetic efficiency, etc (Wu et al., 2007). Yellow-green leaves generally have the same genetic background but show two different leaf colors. Epigenetic modification might play an important role in the formation of leaf color (Li et al., 2015).

Approximately one hundred types of chemical modifications have been reported in eukaryotic RNAs, including N6-methyladenosine (m6A), N1-methyladenosine (m1A), 5-methylcytosine (m5C), 5-hydroxymethylcytosine (hm5C) and inosine (Frye et al., 2016; Zhao et al., 2017; Yang et al., 2020). It is worth noting that m6A modification accounts for 80% of all RNA methylation modifications in eukaryotic cells, and more than 50% of methylated nucleotides in polyA mRNA (Kierzek and Kierzek, 2003; Boccaletto and Bagiński, 2021). In recent years, m6A modification of mRNA in plants has been reported in virous species. m6A methylation could affect many biological processes by interfering with mRNA metabolism, including messenger RNA stability, pre-mRNA splicing, nuclear-to-cytoplasmic export and RNA translation efficiency (Wang X et al., 2014; Wang et al., 2015; Xiao et al., 2016; Roundtree et al., 2017; Liang et al., 2020; Luo et al., 2020; Tang et al., 2023). Recent studies suggested that m6A RNA methylation probably participate in the regulation of the plastid and thylakoid in the leaves (Li et al., 2014; Zhang et al., 2021). However, little is known about the pattern and functions of m6A methylation in regulating leaf color. In order to better understand our biological event, m6A modification will pave the way for further in-depth molecular mechanism analysis.

The m6A modification is regulated by three components: writers (written by methyltransferase), erasers (erased by demethylase), and readers (read by m6A-binding proteins) in plants (Yang et al., 2018; Liang et al., 2020; Zheng et al., 2020; Arribas-Hernandez and Brodersen, 2020; Arribas-Hernandez, 2023). In strawberry, m6A RNA methylation MTA and MTB were highly functionally conserved with dynamic modification of mRNA, and indispensable for fruit ripening (Zhou et al., 2021). In *Arabidopsis thaliana* and *Oryza sativa*, m6A methyltransferase AtFIP37 and OsFIP could influence the fate of shoot stem cells and early degeneration of microspores, respectively (Shen et al., 2016; Zhang et al., 2019). Other m6A demethylases, such as ALKBH2, ALKBH4B, ALKBH8B, ALKBH9B and ALKBH10B, have been discovered that mediate mRNA demethylation to influence the stability of target transcripts, consequently regulating flowering time, trichome and root development, fruit ripening and biotic and abiotic stress responses (Duan et al., 2017; Martínez-Pérez et al., 2017; Zhou et al., 2019; He et al., 2021; Amara et al., 2022; Huong et al., 2022; Han et al., 2023). In addition to methylases and demethylases, a class of m6A readers could fine-regulate biological function. For example, the YTH- domain proteins named EVOLUTIONARILIY CONSERVED C-TERMINAL REGION (ECT). Among which, ECT2, ECT3, ECT4, and ECT13 have been proven to function as m6A readers, playing critical roles in leaf and trichome formation, organogenesis, and nitrate signaling in *Arabidopsis* (Wei et al., 2018; Arribas-Hernandez and Brodersen, 2020; Scutenaire et al., 2018; Hou et al., 2021; Shao et al., 2021; Song et al., 2021). However, it is not clear whether there were other members of these three components involved in m6A modification, and whether these reported components are functionally conserved in different species also needed to be further explored. *Catalpa fargesii* Bur is widely distributed in the middle and western regions, and it is a famous timber and ornamental tree species in China (Wang et al., 2018). Maiyuanjinqiu with yellow-green leaf is a new variety (Identification code: 20150150) that was cultivated from the seedings of *C. fargesii*. The total chlorophyll and photosynthesis were significantly lower in Maiyuanjinqiu than those in *C. fargesi* (Wang et al., 2019). Based on the preliminary experiment, the m6A methylation level of total RNA in yellow leaves was higher than in green leaves. However, the key regulatory mechanism of m6A modifications and transcriptional regulation the leaf color formation was still poorly understood. In our study, transcriptome-wide m6A sequencing was performed in different leaf sectors of Maiyuanjinqiu and *C. fargesii.* Interestingly, the silencing of methyltransferase *CfALKBH5* caused a chlorotic phenotype and increased m6A methylation level, which further confirmed our hypothesis. Our results together indicate that m6A modification is an important epigenetic mark related to leaf color in *C. fargesii* and provide new insights into natural leaf color variation *via* epitranscriptome manipulation in forest breeding.

## Materials and methods

### Plant materials

Maiyuanjinqiu is a variety derived from *C. fargesii* seedlings. The plants samples used in this study were grown in the experimental field of Luoyang, Henan Province in China. All samples were collected, instantly frozen in liquid nitrogen, and stored at -80°C refrigerator. The different leaf color sectors of Maiyuanjinqiu and the responding sectors of *C. fargesii* were divided and collected according to the method of Wang et al. (2019), respectively. For each experiment, at least three biological repeats and technical repetitions were performed.

### RNA extraction

Fresh leaf samples were grinded in mortars with liquid nitrogen, and extracted using RNAprep Pure Plant Plus Kit (TIANGEN, DP441, China), according to the manufacturer’s protocol. Then, the total RNA of the samples was purified with the PrimeScript™ RT Master Mix Kit (TaKaRa, RR036A, China). The concentration and quality of total RNA were tested on a NanoDrop spectrophotometer (Thermo, USA) and gel electrophoresis.

### The quantitative detection of m6A modification

Total RNA isolation and two rounds of PolyA^+^ mRNA selection were performed to measure the global change of the m6A modification level. The change of global m6A levels in mRNA was measured by EpiQuik m6A RNA Methylation Quantification Kit (EpiGentek, P-9005, NY, USA) following the manufacturer’s protocol.

### Library construction, m6A-seq and RNA-seq

The poly(A) mRNAs were fragmented into 100-nucleotide (nt)-long oligonucleotides by using divalent cations. The cleaved RNA fragments were immunoprecipitated by incubating in IP buffer (50 mM Tris-HCl, 0.5% Igepal CA-630, 750 mM NaCl, and 0.5 g/L BSA) at 4 °C for 24 hours with an m6A-specific antibody (Synaptic Systems, Goettingen, Germany). According to the library preparation protocol, immunoprecipitated fragments (IP fractions) and input RNA (input control) libraries were constructed. The Illumina Novaseq 6000 platform was used for RNA sequencing at LC-BIO Biotech (Hangzhou, China). For each experiment, three biological repeats and technical repeats were performed.

### m6A-seq data analyses

m6A-seq and RNA-seq were analyzed following the described previous method (Meyer and Jaffrey, 2014). Firstly, the FastQC tool was used to remove adaptor contamination, low-quality bases, and undetermined bases of raw data (Martin, 2011). Secondly, the clean data were mapped to the reference genome *via* HISAT2 (http://daehwankimlab.github.io/hisat2) (Kim et al., 2015). The mapping reads of input were then used to identify m6A peaks calling *via* the R package (Meng et al., 2014). HOMER and MEME online tools were used to identify conserved sequence motifs using Perl scripts. FPKM (fragments per kilobase of transcript per million mapped reads) was analyzed for the mRNA expression level of genes (Trapnell et al., 2012). The differentially expressed genes were selected with the standard of absolute |Log2FC| ≥ 1 and *P* < 0.05 *via* R package edgeR (Robinson et al., 2010). KEGG enrichment and GO analysis were performed using the LC-BIO online tools (https://www.omicstudio.cn/index).

### qRT-PCR analysis

As described above, total RNAs were extracted from *C. fargesii* leaves. Extracted RNAs were synthesized first-strand cDNA using the PrimeScript**™** RT Reagent Kit (TaKaRa, RR037A, China). qRT-PCR assays were analyzed on the Roche LightCycler**^®^** 480 Real-Time PCR system following the protocol described methods (Wang L et al., 2014). *CfActin* gene was used as internal reference genes (**Supplementary Table S1**) (Wang et al., 2018). The 2^−ΔΔCT^ method was used to analyze the relative mRNA expression levels of genes (Livak and Schmittgen, 2001). For each experiment, three biological repeats and technical repetitions were performed.

### Subcellular localization of CfALKBH5 protein

For the generation of the *CfALKBH5* and GFP fusion gene, the coding region of *CfALKBH5* lacking the stop codon was ligated into the linearized pBI121 vector with the green fluorescent protein (GFP) (**Supplementary Table S2**). The construct *CfALKBH5*-GFP and 35S::GFP (control) were introduced into *Nicotiana benthamiana* lower leaf epidermal cells according to the method of Zheng et al. (2005). After being transformed for 48 h in the dark, the tobacco cells were pelleted, resuspended in infiltrations solution, including 100 μM acetosyringone and 10 mM MgCl_2_, and visualized using a confocal laser scanning microscopy (LSM 700, Zeiss, Jena, Germany).

### VIGS vectors construction and transformation

The full-length coding sequence (CDS) of *CfALKBH5* was cloned using specific primers (**Supplementary Table S2**). The target gene fragment and pTRV2 were digested by *Xba*I and *Sac*I and then ligated by T4 ligase (Promega, M1801, China). The specific vector primers were used to detect the recombinant plasmid pTRV2-*CfALKBH5* by PCR (**Supplementary Table S2**). The constructed vectors were transferred into *Agrobacterium tumefaciens* strain EHA105 by freeze-thaw method (Fire et al., 1998). The *A. tumefaciens* strain was grown at 0.6 OD_600_ with shaking at 120 rpm at 28°C in ILuria-Bertani (LB) medium contained kanamycin (50 mg/L) and rifampicin (50 mg/L). The *A. tumefaciens* strain cells were harvested and re-suspended in an inoculation solution with acetosyringone (200 mM), including 10 mM MgCl_2_, 10 mM MES, and 200 mM acetosyringone. Incubate for 3 hours at room temperature, a mixture (1:1 v/v) of induced EHA105 cultures containing pTRV1 plus pTRV2 vector (control), or pTRV1 plus pTRV2-*CfALKBH5*, was applied using approximately 450 µL of the mixture into the leaves surface of one-month seedlings. At least 3 biological replicates were performed with 15 transgenic plants per replicate. The number of plants for empty vector and untreated control were both same to that of infected plants. Only the most representative individuals were used to photograph. The infected leaves were collected for subsequent experiment analyses.

### Pigment profiling

Fresh leaves were harvested and used to determine total chlorophyll and carotenoid contents following the protocol (Lichtenthaler and Wellburn, 1983). Total chlorophyll and carotenoid contents were extracted with 80% acetone at 4°C overnight in the dark, and then the solution was calculated at 645 nm, 663 nm, and 470 nm against the solvent (acetone) blank with a UV-Vis spectrophotometer (TU-1901, PERSEE, China). For each experiment, three biological repeats and technical repeats were performed.

### Statistical analyses

SPSS19 software was used for statistical analyses, and the statistically significant (*, **, or ***; *P* < 0.05, *P* < 0.01, or *P* < 0.001) were considered as significant differences. For each experiment, three biological repeats and technical repetitions were performed.

## Results

### m6A methylation level is increased in the yellow leaf

**Figure 1:**
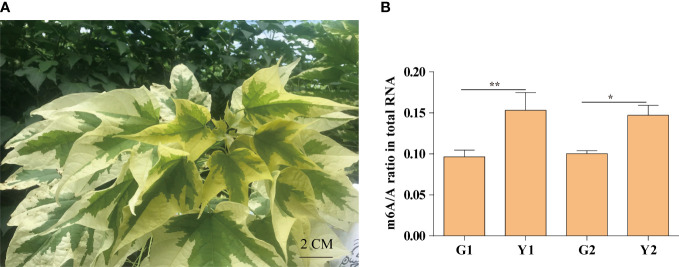
The m6A methylation levels in Maiyuanjinqiu and *C. fargesii*. **(A)** The leaf color character *of* Maiyuanjinqiu. **(B)** The levels of m6A methylation in the leaves of G1, G2, Y1 and Y2 sectors. The asterisks (**, ***) indicate significant differences (*P* < 0.01, *P*<0.001) between the two samples. Bar=2 cm.

Maiyuanjinqiu is a natural variation cultivar derived from *Catalpa fargesii*, whose leaves exhibit yellow-green character (**Figure 1A**). The yellow leaves of Maiyuanjinqiu are named Y1, while the light green leaves in the middle sectores of Maiyuanjinqiu are named Y2. The corresponding sectors of normal green leaves of the *C. fargesii* are named G1 and G2, respectively. To clarify whether m6A methylation is related to leaf color, we examined the overall total RNA m6A methylation levels in different leaf color sectors of Maiyuanjinqiu and the corresponding leaves of *C. fargesii*. As shown in **Figure 1B**, the yellow leaves of Maiyuanjinqiu exhibit hypermethylation compared with the green leaves of *C. fargesii*. The m6A methylation level of yellow leaves reached 1.59 times higher than that in green leaves in Maiyuanjinqiu, while the light green leaves in Maiyuanjinqiu were 1.47 times higher than that in the corresponding green leaves of *C. fargesii*, respectively. Based on the above results, we speculated that a correlation might exist between m6A modifications and leaf color.

### The landscape of m6A methylation in the leaves of Maiyuanjinqiu and *C. fargesii*

**Figure 2:**
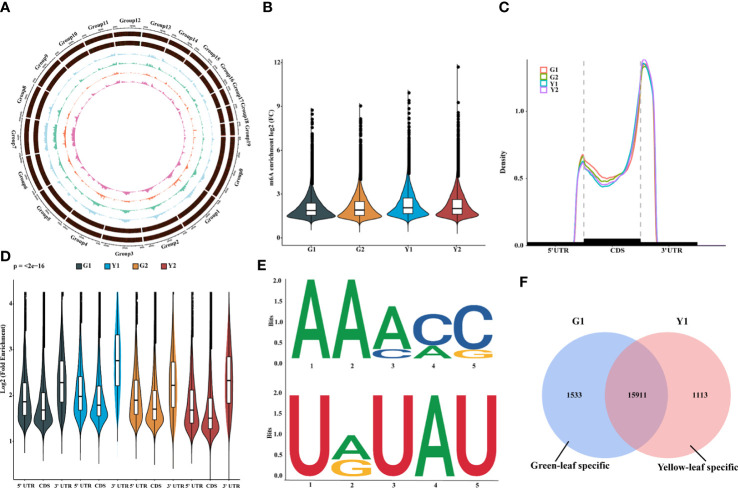
Overview of m6A methylome and transcriptome in leaves between Maiyuanjinqiu and *C.fargesii*. **(A)** Circos plot of the distance and density of m6A peak and expression abundance on *Catalpa bungei* chromosomes. **(B)** Violin plot showed the enrichment of m6A peaks in Maiyuanjinqiu and *Catalpa fargesii*. **(C)** Distribution of m6A peaks in transcript segments divided into 5’-UTR (untranslated region), CDS (coding sequence), and 3’-UTR. **(D)** Violin plot representing a comparison of the fold enrichment of m6A peaks in the different gene segments. **(E)** RRACH and URUAY motifs conserved sequence motifs for m6A-containing peak regions. **(F)** Venn diagram showing the overlap of m6A peaks in the yellow leaves and the corresponding sectors of *C. fargesii*.

To verify our hypothesis, the transcriptome-wide sequencing of m6A methylation was performed with three biological replicates for Maiyuanjinqiu and *C. fargesii* leaves. After eliminating adapter reads, unidentified bases, and low-quality bases, around 67.34%-89.04% of clean reads were uniquely mapped into the *Catalpa bungei* genome (unpublished) (**Supplementary Table  S3**). The identified confident m6A peaks in three replicates with a high Pearson’s correlation coefficient were used for further subsequent analyses (**Supplementary Figure S1**). On average, approximately 30,236 m6A sites of 21,343 genes from G1, around 31,375 m6A sites of 21,429 genes from G2, approximately 32,258 sites of 21,543 genes from Y1, and 32,198 m6A sites of 21,684 genes from Y2 were successfully identified, respectively (**Supplementary Table S4**). The whole-genome density of the m6A peaks and transcripts have different distribution modes mapped on the reference genome (**Figure 2A**). In addition, compared with the leaves of *C. fargesii*, the global hypermethylation of m6A was exhibited in Maiyuanjinqiu leaves (**Figure 2B**).

Furthermore, we discovered that the distribution of m6A peaks in protein-coding mRNA was mostly enriched around the 3’-UTR region in all groups and relatively less in the 5’-UTR (**Figures 2C, D**). In *C. fargesii* leaves, peak callings were almost equally distributed in transcription start (TSS) and end (TES) sites, whereas much more m6A peak callings were discovered in the TES regions than the TSS regions in Maiyuanjinqiu leaves (**Supplementary Figure S2**). Additionally, global m6A peaks were identified two conserved sequences: RRACY (R = G or A; Y = C or U) and UGUAH motifs (H = A, C, or U) using the MEME and HOMER suite (**Figure 2E**), which were very similar to the results identified in rice, tomato, and barley seedlings (Li et al., 2014; Zhang et al., 2019; Miao et al., 2020; Su et al., 2022).

To further understand the relationship between m6A methylation and leaf color, the yellow edge leaves of Maiyuanjinqiu (Y1) and the corresponding green leaves of *C. fargesii* (G1), which have the more representative color difference, were selected for subsequent analysis. We identified 1,533 green-leaf specific m6A peaks and 1,113 yellow-leaf specific peaks, as well as 15,911 common peaks (**Figure 2F**). KEGG enrichment analysis showed that m6A-modified genes unique to green leaves are mainly involved in flavone and flavonoid biosynthesis, anthocyanin biosynthesis, and starch and sucrose metabolism (**Supplementary Figure S3A**). However, m6A-harboring genes specific to yellow leaves are principally involved in photosynthesis-antenna proteins and phenylalanine metabolism (**Supplementary Figure S3B**).

### The m6A levels are slightly negatively correlated with global gene expression

**Figure 3:**
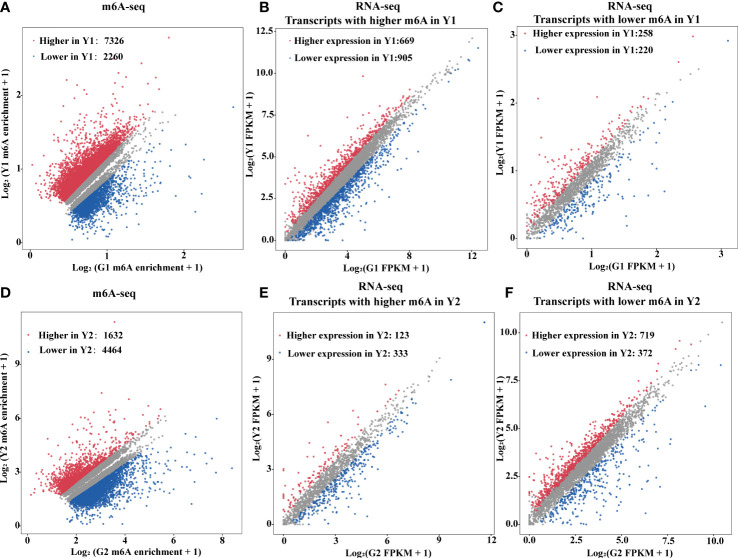
m6A RNA methylation is negatively associated with gene abundance. **(A)** Scatter plots exhibited transcripts abundance with differential m6A enrichment between the yellow leaves (Y1) and the corresponding green leaves (G1). The m6A-modified transcripts with substantially higher and lower peak enrichment in Y1 compared to G1 are highlighted in red and blue, respectively (|Log_2_FC| ≥ 1; *P* value < 0.05). **(B)** Expression of m6A-modified transcripts with markedly higher peak enrichment in Y1 than in G1. **(C)** Expression of m6A-modified transcripts with markedly lower peak enrichment in Y1 than in G1. **(D)** Scatter plots exhibited transcripts abundance with differential m6A enrichment between light green leaves (Y2) and the corresponding green leaves (G2). The m6A modified transcripts with substantially higher and lower peak enrichment in Y2 compared to G2 are highlighted in red and blue, respectively (|Log_2_FC| ≥ 1 and P < 0.05). **(E)** Expression of m6A-modified transcripts with markedly higher peak enrichment in Y2 than in G2. **(F)** Expression of m6A-modified transcripts with markedly lower peak enrichment in Y2 than in G2. RNA-seq was used for gene expression analysis. FPKM, fragments per kilobase of exon per million mapped fragments.

m6A deposition has been reported to influence mRNA abundance (Shen et al., 2016; Wang X et al., 2014; Duan et al., 2017; Zhao et al., 2017). To estimate the potential correlation between m6A mRNA methylation and gene expression during leaf yellowing, m6A-seq and RNA-seq were analyzed for the enhancement in the levels of m6A-containing transcripts and the global gene expression changes between two samples (**Figure 3**). A total of 9,586 transcripts with differential m6A levels (log2(FC) ≥1, *P* value < 0.05) between Y1 versus G1 were identified. Compared with Y1, 7,326 transcripts had higher m6A methylation levels than G1, while 2,260 transcripts had lower m6A methylation levels than G1 (**Figure 3A**). Among the 7,326 transcripts, only 669 showed higher expression levels, whereas 905 exhibited lower expression levels in Y1 versus G1 groups (**Figure 3B**). Accordingly, 719 and 372 displayed higher and lower expression levels among the 4,464 transcripts with lower m6A levels in Y2 compared to that in G2, respectively (**Figure 3F**, **Supplementary Table S5**). These data suggested that m6A methylation was slightly negatively correlated with the expression of the global transcripts.

### Transcriptional differences in different leaf color sectors

**Figure 4:**
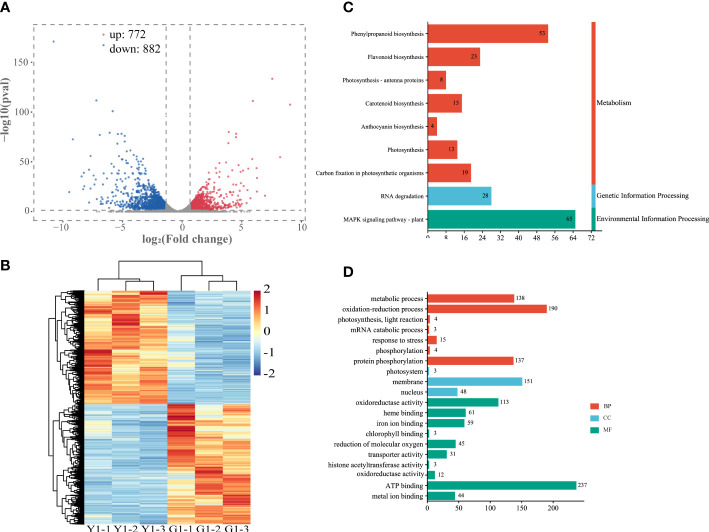
Analysis of differentially expressed genes (DEGs) in the leaves of Y1 and G1. **(A)** Volcano plot and Venn graph of regulated genes. **(B)** Heatmap showed the correlation of transcript expression between three biological replicates between Y1 and G1. **(C)** KEGG enrichment pathways of the DEGs between Y1 and G1. **(D)** GO enrichment of DEGs. BP, biological process; CC, cellular component; MF, molecular function.

To further explore the relationship between gene expression and leaf color, the yellow leaves (significant phenotypic characteristics) and the corresponding green leaves were selected for transcriptome differential analysis. A total of 3,308 differentially expressed genes (DEGs) were analyzed by the comparison in Y1 versus G1 group, with 772 up-regulated genes and 882 down-regulated genes, respectively (**Figure 4A**, **Supplementary Table S6**). Heatmap plot of the differentially expressed genes showed the consistency of three biological replicates and the significant difference between yellow and green leaves, respectively (**Figure 4B**). KEGG analysis showed that all significant DEGs were enriched in 129 metabolic or biological pathways. Remarkably, DEGs were highly enriched in the pathways of flavonoid biosynthesis, photosynthesis-antenna proteins, carotenoid biosynthesis, photosynthesis, anthocyanin biosynthesis, and RNA transport (**Figure 4C**). GO analysis revealed these DEGs are highly enriched in response to photosynthesis, light reaction, response to stress, heme binding, chlorophyll metabolism, and metal ion binding (**Figure 4D**). These results showed that the mRNA expression levels of photosynthesis, pigment, ion-binding and stress-related genes contributed to the formation of leaf color in Maiyuanjinqiu.

### Correlation analysis of m6A-modified genes and DEGs expression on in yellow and green leaves

**Figure 5:**
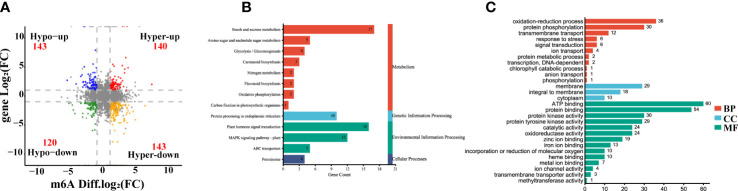
Integrating analysis of differentially m6A-modified and expressed transcripts between the yellow leaves (Y1) and the corresponding green leaves (G1). **(A)** Four-quadrant diagram exhibiting a relationship between m6A methylation and transcripts expression. Hyper-up, hypermethylation and up-regulated genes. Hyper-down, hypermethylation and down-regulated genes. Hypo-up, hypomethylation and upregulated genes. Hypo-down, hypomethylation and down-regulated genes. **(B)** KEGG pathways of differentially expressed genes (DEGs) with m6A peaks. **(C)** GO enrichment of DEGs with m6A peaks. MF, Molecular Function; CC, Cellular Component; BP, Biological Process.

According to the crosstalk analysis of the m6A-seq and RNA-seq, we found 546 DEGs had differentially expressed peaks (DPs) in the comparison between yellow leaf and leaves. Among them, 140 transcripts showed m6A hypermethylation and up-regulated expression (Hyper-up), 143 transcripts displayed m6A hypermethylation and down-regulated expression (Hyper-down), 143 transcripts exhibited m6A hypomethylation and up-regulated expression (Hypo-up), and 120 transcripts had m6A hypomethylation and down-regulated expression (Hypo-down) (**Figure 5A**, **Supplementary Table S7**). Furthermore, KEGG analysis of those yellow leaf yellow genes in the four-quadrant plot revealed that they were abundant in 69 pathways, including plant hormone signal transduction, peroxisome, porphyrin and chlorophyll metabolism, carotenoid biosynthesis, flavonoid biosynthesis and carbon fixation in photosynthetic organisms (**Figure 5B**). Meanwhile, GO analysis revealed that those genes were highly enriched in the oxidation-reduction process, response to stress, heme binding and metal ion binding (**Figure 5C**).

### CfALKBH5 is a putative demethylase for m6A RNA that contributes to the yellow leaves

**Figure 6:**
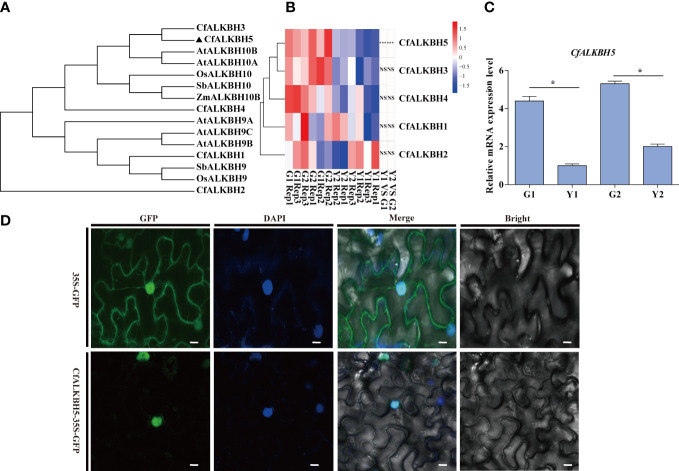
Phylogenetic tree construction, gene expression analyses and subcellular localization of m6A RNA demethylases ALKBHs. **(A)** The phylogenetic tree of ALKBHs in *C. fargesii* and other ALKBH proteins by the neighbor-joining method using MEGA 6. The sequences of the ALKBH proteins were achieved from the NCBI website (https://www.ncbi.nlm.nih.gov/protein/), and species names are shown below. Species names are abbreviated as follows: Cf, *C. fargesii*; At, *Arabidopsis thaliana*; OS, *Oryza sativa L*; Sb, *Sorghum bicolor*; Zm, *Zea mays.* **(B)** Heatmap analysis revealing the gene expression of *CfALKBH1-5* in G1, G2, Y1 and Y2 sectors based on RNA-seq. Data are presented as the mean of three independent biological replicates. **(C)** The relative mRNA expression of *CfALKBH5* in yellow leaves and green leaves as determined by qRT-PCR analysis. Asterisks indicate significant differences (*P < 0.05). **(D)** Subcellular localization of CfALKBH5 in tobacco leaf epidermal cells. The 35S::GFP and construct for 35S::CfALKBH5-GFP plasmid was introduced into tobacco epidermal cells by particle bombardment. The nuclei of the tobacco leaf epidermal cells were detected *via* DAPI staining. Bar=20 μm.

A large number of differential sites of m6A methylation modification were identified (**Figure 5A**), and the methylation level in yellow leaves was higher than that of green leaves. The differences in m6A levels might result from the regulation of m6A methyltransferases and demethylases. According to the existing literature report, the m6A writers mainly perform biological functions through a complex composed of methyltransferase METTL3 and METTL14 (Shi et al., 2019).The m6A writers mainly perform biological functions through a complex composed of methyltransferase METTL3 and METTL14 (Shi et al., 2019). The homologous genes of *METTL3* and *METTL14* were identified in the genome of *C. fargesii*, named *CfMTA1* and *CfMTA2*, respectively. And both of which contained a conserved MT-A70 domain (**Supplementary Figure S4A**). Based on the RNA-seq, the mRNA expression levels of *CfMTA1* and *CfMTA2* did not change significantly in the yellow leaves (**Supplementary Figure S4B**). It is speculated that m6A methyltransferase might not be an important reason for the overall change of m6A methylation modification level in yellow leaves. However, five demethylase *ALKBHs*, from *CfALKBH1* to *CfALKBH5*, were markedly down-regulated in the yellow leaves. The phylogenetic tree showed that CfALKBH3 and CfALKBH5 shared high similarity with AtALKBH10A and AtALKBH10B of *Arabidopsis* (**Figure 6A**). However, only the expression level of *CfALKBH5* was remarkably decreased in the yellow-green leaves compared with that of *C. fargesii* among the five *CfALKBH* genes based on RNA-seq analysis results (**Figure 6B**, **Supplementary Table S8**). This result was further confirmed by qRT-PCR assay (**Figure 6C**). Therefore, *CfALKBH5* gene might regulate gene expression by regulating m6A methylation, and thus affect leaf color.

### CfALKBH5 is a nuclear protein

To further investigate the nucleus localization of CfALKBH5, the plasmid encoding the 35S::CfALKBH5-GFP and 35S::GFP control were transiently expressed in tobacco epidermal cells. The fluorescence signals of CfALKBH5-GFP were localized in the nucleus of epidermal cells, whereas 35S-GFP was detected to be uniformly distributed throughout the tobacco epidermal cells (**Figure 6D**). These data indicated that CfALKBH5 was a nuclear-localized protein.

### The suppression *CfALKBH5* results in a chlorotic phenotype in *C. fargesii*

**Figure 7:**
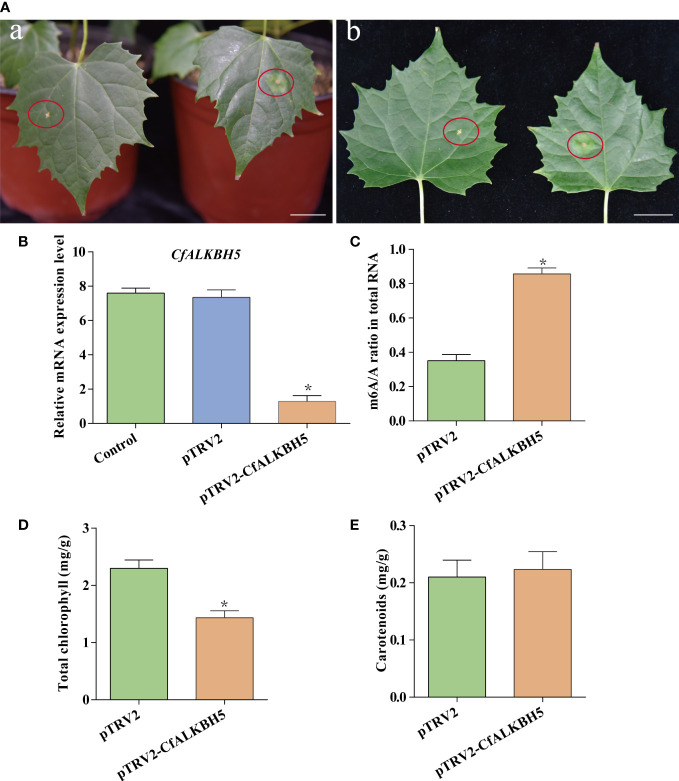
The function analysis of a putative methyltransferase CfALKBH5 by silencing *CfALKBH5* gene *in C.fargesii*. **(A)** VIGS-mediated CfALKBH5-silenced plants (**Figure 7A**) compared to pTRV2 vector-infected plants (control, **Figure 7B**). 15 plants were selected for photography in each treatment, and the most representative photos were selected. The red circle represents the infected area. Bar=2 cm. **(B)** The expression levels of *CfALKBH5* in pTRV2-*CfALKBH5* and control plants. The relative expression levels are shown as fold change values. Asterisk indicates a significant difference (*, *P*< 0.05). **(C)** The levels of m6A in total RNA of pTRV2- and pTRV2- *CfALKBH5*-treated plants, respectively. **(D, E)** The total chlorophyll and carotenoid contents were measured in the leaves of pTRV2-*CfALKBH5* and control plants.

The pTRV2-*CfALKBH5* (suppression) and pTRV2 vector (empty vector) were infected using an efficient virus-induced gene silencing (VIGS) system following to previous protocol (Chen et al., 2017; Yang et al., 2020), which has been generally used in functional characterization of genes in plenty of plant species (Chen et al., 2017; Yang et al., 2020). To ensure the accuracy of this experiment, a minimum of three biological replicates were performed, which contain at least 15 transformed *C. fargesii* plants in each replicate. A chlorotic phenotype was observed in pTRV2-*CfALKBH5*-infected plants (**Figure 7A**, **Supplementary Figure S5**). The relative mRNA expression level of *CfALKBH5* was significantly lower in pTRV2-*CfALKBH5* infected plants than that in control plants, indicating the suppression was effective in our experiment (**Figure 7B**). Furthermore, the methylation level was detected by the m6A methylation kit, the result showed that *CfALKBH5* silencing significantly increased the m6A level in the total RNA (**Figure 7C**). The contents of chlorophyll and carotenoid were analyzed in the leaves of *CfALKBH5*-silenced and control seedlings. Compared with the control plants, the total chlorophyll content of leaves markedly decreased in *CfALKBH5* silencing plants, but the content of carotenoid did not significantly change (**Figures 7D, E**). The results showed that *CfALKBH5* might play a role in chlorophyll synthesis/metabolism by regulating the methylation levels.

### The suppression of *CfALKBH5* could change the expression level of pigment biosynthesis and photosynthesis genes

**Figure 8:**
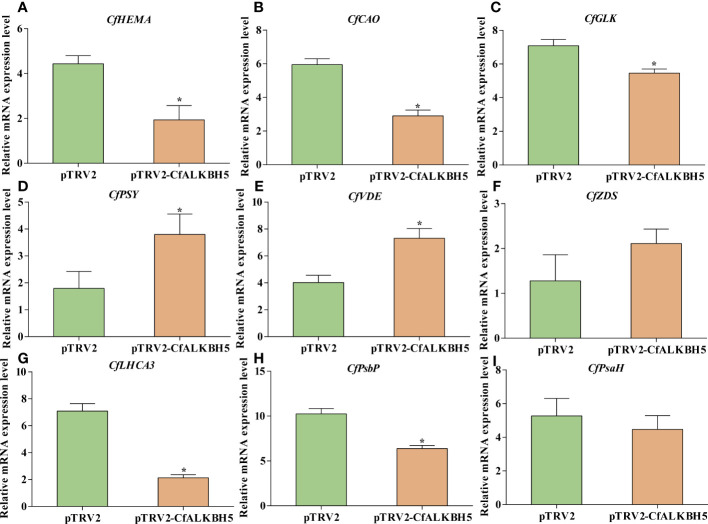
The expression comparison of important genes involved in chlorophyll metabolism, carotenoid biosynthesis and photosynthesis between pTRV2-*CfALKBH5*-and pTRV2-infected plants. **(A–C)** The expression levels of *CfHEMA*, *CfCAO*, *CfGLK* genes involved in chlorophyll metabolism in pTRV2-*CfALKBH5*-and pTRV2-infected plants. **(D–F)** The expression levels of *CfPSY*, *CfVDE*, *CfZDS* genes involved in carotenoid biosynthesis in pTRV2-*CfALKBH5*-and pTRV2-infected plants. **(G–I)** The expression levels of *CfLHCA3*, *CfPsbP*, *CfPsbH* genes involved in photosynthesis chlorophyll metabolism in pTRV2-*CfALKBH5*-and pTRV2-infected plants. Asterisks represent the significant difference, *P* < 0.05.

Based on m6A-seq and RNA-seq data, the m6A levels of some key genes involved in chlorophyll metabolism, carotenoid biosynthesis, and photosynthesis were altered in yellow leaves (**Supplementary Table S9**). Therefore, qRT-PCR was performed to explore whether these genes also changed in pTRV2-*CfALKBH5*-infected and control plants. Compared with the control leaves, the expression levels of chlorophyll biosynthesis-related genes *CfHEMA*, *CfCAO* and *CfGLK* and photosynthesis-related genes *CfLHCA3* and *CfPsbP* were down-regulated in pTRV2-*CfALKBH5*-infected plants (**Figure 8**). In contrast, the gene expression of *CfPSY* and *CfVDE* involved in carotenoid synthesis were up-regulated in the *CfALKBH5*-silenced leaves (**Figure 8**). Taken together, *CfALKBH5* suppression indeed induced the change of gene expression profiles in pigment biosynthesis and photosynthesis.

## Discussion

m6A methylation is an important mechanism of epigenetic regulation at the post-transcriptional level and has been studied in eukaryotic, including mice, human, Arabidopsis, rice, barley, tomato, sea buckthorn and apple (Meyer and Jaffrey, 2014; Zhao et al., 2014; Zhang et al., 2016; Zhou et al., 2019; Hu et al., 2021; Zhang et al., 2021; Hou et al., 2022; Su et al., 2022). However, as a valuable horticultural tree, the abundance, distribution, and function of m6A modification of *C. fargesii* remain unclear. In this research, we confirmed the presence of m6A modification of yellow-green leaves in *C. fargesii*. Firstly, we used an m6A RNA methylation quantification kit to detect the presence differences of m6A methylation levels in different color leaves of Maiyuanjinqiu and *C. fargesii*. Secondly, we identified the m6A methylation sites in *C. fargesii* genome based on Illumina Novaseq sequencing. In addition, m6A methylation levels presented differences in the total RNA between Maiyuanjinqiu and *C. fargesii*, indicating functional diversity of m6A modification and providing new insights into early post-transcriptional regulation during leaf chlorosis.

The transcriptome-wide distribution of m6A methylation is divergent in eukaryotes, including transposable element gene exons and transposons (Hu et al., 2021; Song et al., 2021). In this research, m6A-seq revealed m6A distribution pattern mostly in the 3’-UTR of mRNAs both in Maiyuanjinqiu and *C. fargesii* (**Figure 2C**), which is consistent with previous reports that have demonstrated m6A peaks near the 3’-UTR in plants (Shen et al., 2016; Zhang et al., 2019; Zhou et al., 2019; Luo et al., 2020; Hu et al., 2021), Notably, we discovered highly conserved RRACH motif and URUAY (R = A/G, Y = U/A) sequence as a previously identified plant-specific motif (Wei et al., 2018; Zhou et al., 2019; Luo et al., 2020; Hu et al., 2021). In addition, RRACH-like motifs, which were found in humans, mice, Arabidopsis, and maize (Dominissini et al., 2013; Meyer et al., 2012; Linder et al., 2015; Duan et al., 2017; Miao et al., 2020) (**Figure 2E**). In addition, our findings revealed an overall negative association between mRNA m6A modification and transcripts abundance (**Figure 3**), which was consistent with the findings of the ripening of tomato fruits (Zhou et al., 2019). Moreover, we found several genes with m6A modification are related to photosynthesis, which is similar to those in *Arabidopsis* (Li et al., 2014). The above results indicated that m6A modifications were conservative in plants. Unexpectedly, there are more increased m6A-methylated genes in Y1 but more decreased m6A-methylated genes in Y2. We speculated that the formation mechanisms of the natural yellow leaves are complicated, and there might be other underlying compensation mechanism might play a role in the light green leaf (Y2) of Maiyuanjinqiu. For example, in addition to demethyltransferase ALKBH5, whether there were other unknown m6A methyltransferases might be also involved in regulating the process deserve further investigation.

Several studies have demonstrated that RNA demethylases and methyltransferases may bind to and remove m6A marks, and play a vital role in regulating mRNA fate (Hu et al., 2019; Huang et al., 2020; Shao et al., 2021). In particular, several YT521-B homology (YTH) domain-containing proteins have been identified as m6A readers that regulate either negative or positive mRNA stability in animals and plants (Du et al., 2016; Shi et al., 2017; Huang et al., 2018; Wei et al., 2018; Baquero-Perez et al., 2019; Song et al., 2021). Moreover, several studies have demonstrated that the physiological functions of RNA methyltransferases were illustrated in different species. For example, the activity of mRNA methyltransferase METTL3 was influenced by SUMOylation in mammals (Du et al., 2018). In *Arabidopsis*, five potential RNA demethylases were identified, among which, ALKBH10B was involved in the floral transition and abiotic stress, while ALKBH9B participated in alfalfa mosaic virus infection (Duan et al., 2017; Martínez-Pérez, 2017; Han et al., 2023). In this study, an extensive search of the *Catalpa bungei* genome was performed and identified five putative RNA demethylases. Based on the transcriptome and qRT-PCR results, the expression levels of *CfALKBH5* reduced dramatically in yellow-green leaves of Maiyuanjinqiu compared with those of *C. fargesii*. In this study, CfALKBH5 nuclear localization suggested that CfALKBH5 is mainly responsible for nuclear RNA methylation rather than chloroplast or mitochondrial RNA methylation. In tomato, SlALKBH2 localized to the endoplasmic reticulum (ER) and regulated fruit ripening (Zhou et al., 2019). In *Arabidopsis*, CPSF30 localized to the nucleus and regulated the splicing of mRNA implicated in the salicylic acid pathway in response to external stimuli (Bruggeman et al., 2014). The localization of methylase might be related to its potential function, the functional analysis of *CfALKBH5* is very important for biological issues of concern.

In this study, the silencing of *CfALKBH5* significantly increased the abundance of m6A modification in total RNA in *C. fargesii*, indicating that CfALKBH5 indeed influences m6A modification. Moreover, CfALKBH5 suppression resulted in a chlorotic leaf phenotype and decreased chlorophyll contents. Moreover, qRT-PCR results showed that the expression levels of several critical genes involved in chlorophyll biosynthesis, carotenoid biosynthesis and photosynthesis had changed in pTRV2-*CfALKBH5*-infected plants. It is worth considering that although we detected changes of the expression levels of *CfPSY* and *CfVDE* genes in *pTRV2-CfALKBH5*-infected plants, there was no significant difference in carotenoid content. This means that more complex regulatory mechanisms might be involved in this process except for m6A methylation, which needed to be further studied. For example, whether these genes involved in chlorophyll metabolism, carotenoid biosynthesis, and photosynthesis are m6A modified and whether the m6A levels of these genes are altered in *CfALKBH5* knockdown plants remain unclear. In addition to m6A methylation, are there other types of modification regulating leaf color of Maiyuanjinqiu also need to be studied.

## Conclusion

In summary, we first investigated the differences of global m6A methylation levels in different leaf color sectors in woody plants. We found that the m6A methylated sites were mainly identified around the 3’-untranslated regions (3’-UTR), which was slightly negatively correlated with the mRNA abundance. Furthermore, the m6A methylation levels were significantly enhanced in the yellow sectors compared with the green sectors based on m6A RNA methylation quantification detection and m6A-seq data. Crosstalk analyses between peak and differential genes were conducted, KEGG and GO analyses showed that m6A modification genes were associated with photosynthesis, pigments biosynthesis and metabolism, oxidation-reduction and response to stress, etc. In addition, the overall increase in m6A methylation is associated with the decreased expression of RNA demethylase gene *CfALKBH5*. Interestingly, the silencing of demethylase *CfALKBH5* caused a chlorotic phenotype and increased m6A methylation level, suggesting the role of *CfALKBH5* in the formation of yellow-green leaves of Maiyuanjinqiu. Our results revealed that m6A modification could be considered as a vital epigenomic mark and contribute to the naturally variations in plants.

## Data availability statement

The data presented in the study are deposited in the National Genomics Data Center repository (https://ngdc.cncb.ac.cn/gsa/browse/CRA010119), the accession number is CRA010119. The datasets generated and /or analyzed during the current study are available in NCBI with the accession number OQ401832 (https://www.ncbi.nlm.nih.gov/search/all/?term=OQ401832). Further inquiries can be directed to the corresponding author.

## Author contributions

YZ performed the experiments and wrote the original draft. NW conceived the experiment and revised the paper. PF, WM and NL contributed to data analysis. YY, LZ, JH and GQ provided help in the experiments. JW provided the project funds, supervised the entire experiment and writing process. All authors contributed to the article and approved the submitted.
