# Supplementary material for: Transcriptome-wide m6A methylation in natural yellow leaf of Catalpa fargesii
Source: Front Plant Sci. 2023 Jun 19;14:1167789. doi: 10.3389/fpls.2023.1167789 (PMC10315917; doi:10.3389/fpls.2023.1167789)
Supplement: Supplementary file 1 [file Image_1.pdf]

Supplementary Figures

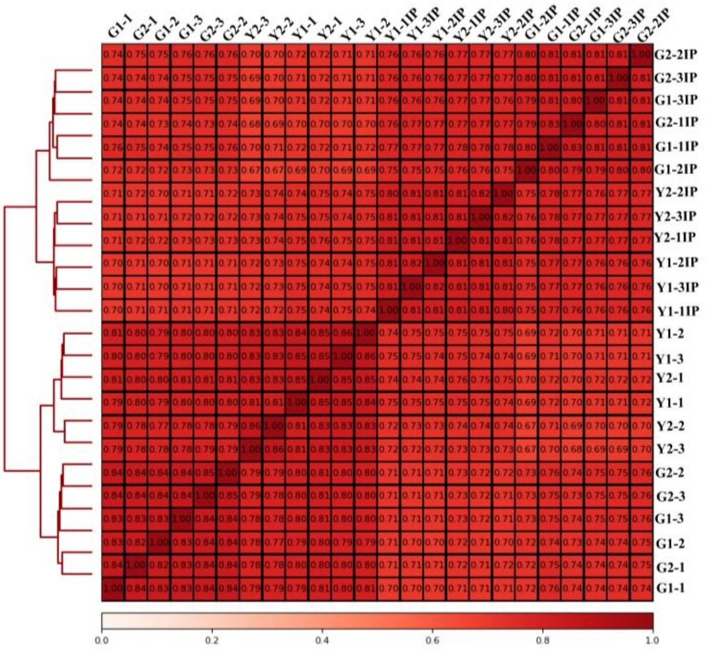

Figure S1. Pearson correlation of m6A peak among three biological replicates under *Maiyuanjinqiu* and *C. fargesii* leaves. m6A peak abundances are normalized as log10 (RPKM). IP: immunoprecipitation using m6A antibody.

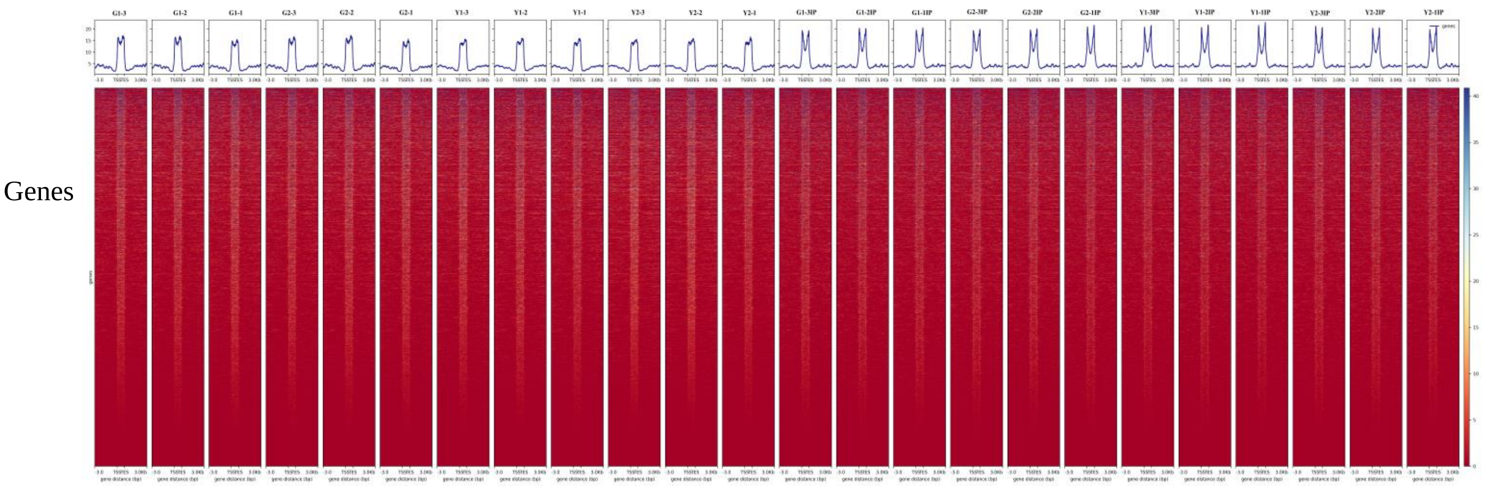

Figure S2 Enrichment of m6A peaks near the transcription start site (TSS) and the transcription end site (TES) of genes. The degree of peak enrichment is represented by heatmap and peak plots.

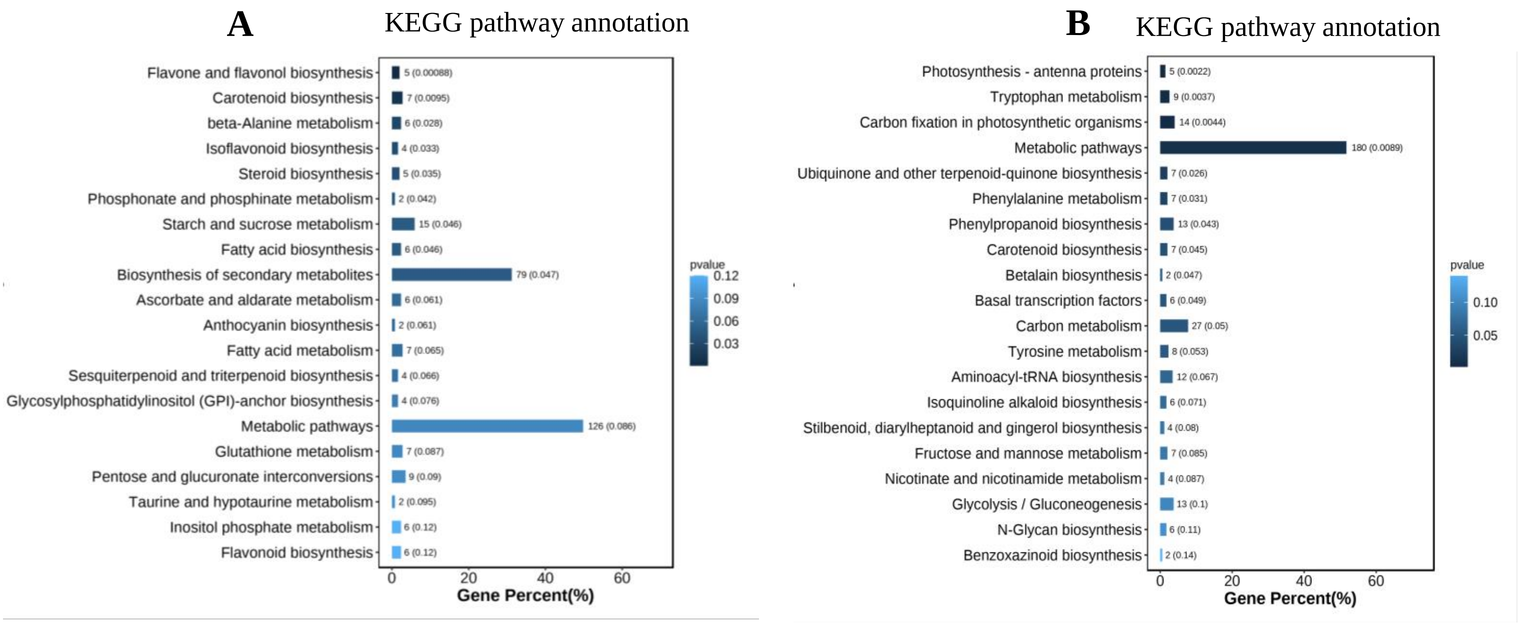

Greenleaf - specific **Yellowleaf-specific**

Figure S3 KEGG analysis of the differently greenleaf-specific m6A-containing peaks and yellowleaf-specific m6A-containing peaks.

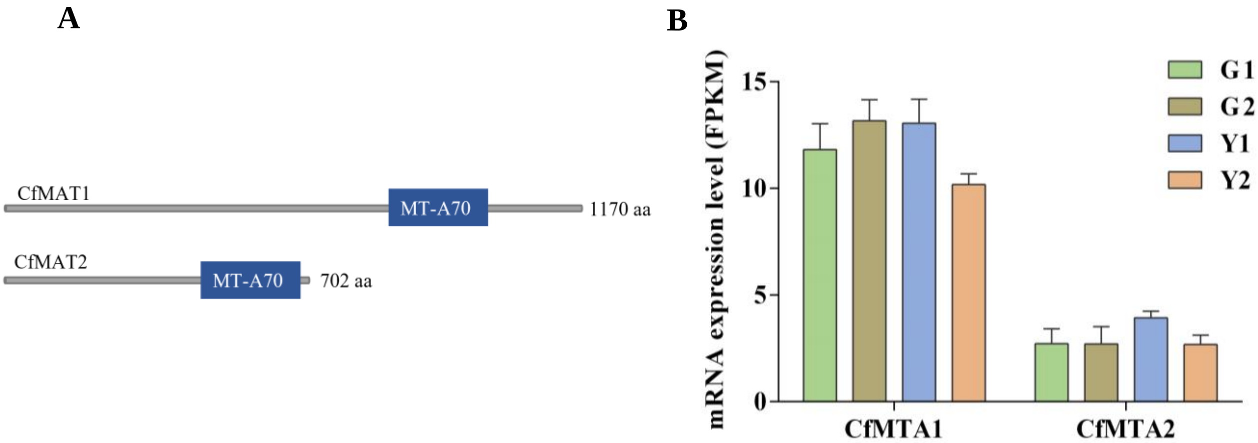

Figure S4 The mRNA expression of potential m6A methyltransferases MTA in *C. fargesii*

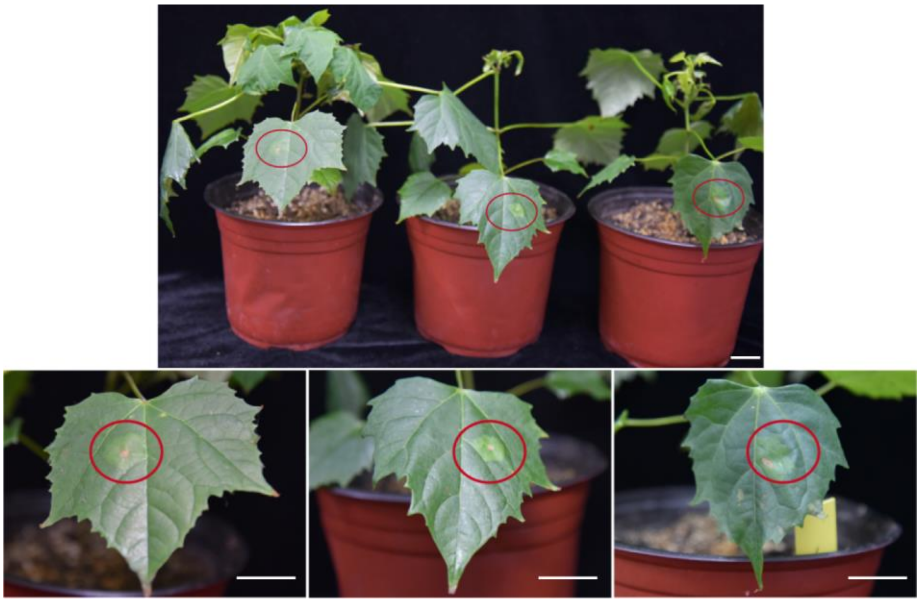

Figure S5 VIGS-mediated CfALKBH5-silenced plants. The red circle represents the infected area. Bar=2 cm
